# Preoperative Salivary Active MMP-8 and Early Dental Implant Stability Trajectories: A Prospective Cohort Study

**DOI:** 10.3390/jcm15218650

**Published:** 2026-11-06

**Authors:** Adel Alenazi

**Affiliations:** Department of Oral and Maxillofacial Surgery and Diagnostic Sciences, College of Dentistry, Prince Sattam Bin Abdullaziz University, Al-Kharj 16273, Saudi Arabia; a.alenazi@psau.edu.sa

**Keywords:** active MMP-8, dental implants, implant stability, saliva, resonance frequency analysis, biomarker, prospective cohort

## Abstract

**Objectives:** We aim to investigate whether preoperative salivary active matrix metalloproteinase-8 (aMMP-8) is associated with the early dental implant-stability trajectory during the first 12 weeks after implant placement. **Materials and Methods:** This prospective, single-center cohort study included 125 adults receiving one implant each in healed edentulous sites. Unstimulated whole saliva was collected preoperatively for aMMP-8 assessment. Implant stability was assessed by resonance frequency analysis and expressed as mean implant stability quotient (ISQ) at placement (T0) and 4, 8, and 12 weeks. Longitudinal ISQ was analyzed using an unstructured-covariance mixed model for repeated measures with categorical time, adjusted for age, sex, smoking, diabetes status, periodontal status, salivary flow, jaw, implant dimensions, insertion torque, and bone quality. The primary analysis evaluated the global 3-df aMMP-8 × time interaction, followed by Holm-adjusted week-specific contrasts. **Results:** Of 500 scheduled ISQ measurements, 471 (94.2%) were observed. Six of 125 implants (4.8%) failed during the 12-week follow-up. Mean ISQ decreased from 75.9 ± 8.3 at T0 to 69.8 ± 7.5 at week 4 and subsequently increased to 71.4 ± 6.7 and 73.8 ± 7.6 at weeks 8 and 12, respectively. There was evidence of an aMMP-8 × time interaction (*F*(3, 112.1) = 8.81; *p* < 0.001). Each 1-SD higher preoperative ln(aMMP-8), equivalent to approximately a 1.89-fold higher concentration, was not clearly associated with ISQ change from T0 at week 4 (−0.81 ISQ units; 95% CI, −1.85 to 0.23; Holm-adjusted *p* = 0.126), but was associated with less favorable ISQ change at week 8 (−2.55; 95% CI, −3.75 to −1.35; Holm-adjusted *p* < 0.001) and week 12 (−3.09; 95% CI, −4.39 to −1.79; Holm-adjusted *p* < 0.001). The adjusted association at T0 was close to null (0.30; 95% CI, −1.07 to 1.66; *p* = 0.669), and the week-8 and week-12 findings were generally consistent across conventional sensitivity analyses, although the week-12 association was attenuated and no longer statistically significant under some failure-inclusive assumptions. **Conclusions:** Higher preoperative salivary aMMP-8 was associated with a less favorable early implant-stability trajectory, with differences emerging at 8 weeks and persisting at 12 weeks in the primary analysis. These findings support further evaluation of salivary aMMP-8 as a non-invasive preoperative marker of variability in early implant healing, although the clinical significance of the observed ISQ differences and their implications for implant outcomes remain uncertain.

## 1. Introduction

Successful dental implant therapy depends, in part, on achieving adequate implant stability at placement and maintaining a favorable stability profile during early healing [1,2]. Implant stability is a dynamic construct comprising primary stability, which is predominantly determined by mechanical engagement between the implant and surrounding bone at insertion, and secondary stability, which progressively develops through bone healing and remodeling at the bone–implant interface [2,3]. During the transition between these phases, primary stability may decline as biologically mediated secondary stability develops, producing characteristic temporal changes in implant stability during the early postoperative period [2,4]. Resonance frequency analysis (RFA), expressed as the implant stability quotient (ISQ), provides a non-invasive and repeatable approach for quantifying implant stability and enables longitudinal monitoring during healing [1,3,5]. However, ISQ reflects the mechanical stability of the implant–tissue complex rather than a direct histological measure of osseointegration, and its trajectory is influenced by multiple implant-, site-, and healing-related factors [3,5]. Consequently, serial assessment of implant stability provides a framework for characterizing the early healing course and investigating biological factors associated with interindividual variation in that stability trajectories [4,5].

Early implant healing involves coordinated inflammatory, extracellular-matrix, and bone-remodeling responses, suggesting that host-response biomarkers may provide biological information complementary to conventional mechanical measures of implant stability [6]. Among these biomarkers, matrix metalloproteinase-8 (MMP-8), also known as neutrophil collagenase or collagenase-2, is a zinc-dependent proteolytic enzyme involved in extracellular-matrix turnover and is released predominantly by activated neutrophils during inflammatory responses [7,8]. Importantly, MMP-8 exists in latent and activated forms, and its enzymatically active form (aMMP-8) reflects ongoing proteolytic activity more directly than measurements that do not distinguish between latent and active MMP-8 [7,8,9]. Elevated aMMP-8 concentrations in oral fluids, including saliva, mouth rinse, gingival crevicular fluid, and peri-implant sulcular fluid, have been associated with periodontal and peri-implant inflammatory conditions and investigated as biomarkers for disease detection and monitoring [7,8,9,10]. Recent evidence further supports the diagnostic potential of aMMP-8 in peri-implant disease, although methodological heterogeneity and the need for longitudinal validation remain important limitations [10]. Because secondary implant stability develops through inflammatory resolution, extracellular-matrix remodeling, and new bone formation at the bone–implant interface, the preoperative host inflammatory state may be associated with subsequent stability changes. Elevated salivary aMMP-8 may therefore reflect a collagenolytic inflammatory profile associated with variation in early healing rather than primary mechanical fixation. Accordingly, preoperative salivary aMMP-8 may provide a non-invasive indicator of the host inflammatory and tissue-remodeling milieu that is biologically distinct from the mechanical information captured by implant stability measurements.

Despite growing interest in biological markers of peri-implant tissue status, their relationship with the dynamic course of implant stability during early healing remains incompletely characterized. Previous prospective studies have used serial RFA to characterize early implant-stability changes and explored associations between implant stability and local bone-turnover markers during healing [11,12,13]. However, the available aMMP-8 literature in implant dentistry has predominantly focused on peri-implant sulcular fluid measurements for the detection or monitoring of established peri-implant inflammation and disease [10,14], rather than on the host-response state before implant placement. Consequently, it remains unclear whether preoperative salivary aMMP-8 is associated with the subsequent trajectory of implant stability during early healing. Addressing this question requires a prospective design in which the biomarker is measured before surgery and implant stability is repeatedly assessed thereafter, thereby preserving the temporal sequence between the preoperative host-response profile and the subsequent stability trajectory.

Clarifying this relationship may have clinical relevance because a non-invasive preoperative host-response marker could complement established mechanical and site-related assessments by providing information on biological variation related to the subsequent course of implant healing. Such information may ultimately inform the development of individualized monitoring strategies, although prognostic performance and clinical utility would require dedicated validation. Therefore, this prospective cohort study aimed to investigate whether preoperative salivary aMMP-8 was associated with the early dental implant-stability trajectory, assessed by serial ISQ measurements from implant placement through 12 weeks of healing. We hypothesized that longitudinal changes in ISQ would vary according to preoperative salivary aMMP-8 levels.

## 2. Materials and Methods

### 2.1. Study Design and Settings

This prospective, single-center observational cohort study was conducted at the Oral and Maxillofacial Surgery Department, Faculty of Dentistry, Alexandria University, Alexandria, Egypt. Adults scheduled to receive a single endosseous dental implant in a healed edentulous site were prospectively recruited between January 2024 and February 2025 and followed from preoperative assessment through 12 weeks after implant placement. Each participant contributed one study implant. Preoperative salivary active matrix metalloproteinase-8 (aMMP-8) was assessed before implant placement, and implant stability was evaluated by resonance frequency analysis (RFA) at implant placement (T0) and at 4, 8, and 12 weeks. The study was approved by the Institutional Review Board of the Faculty of Dentistry, Alexandria University, Egypt, and the Research Ethics Committee, Alexandria Faculty of Dentistry (IRB 00010567; IORG 0008939). All participants provided written informed consent, and study procedures were conducted in accordance with the Declaration of Helsinki. Reporting followed the STROBE statement and STROBE-ME recommendations.

### 2.2. Study Population

Adults (≥18 years) scheduled to receive a single endosseous dental implant in a healed edentulous site were prospectively screened for eligibility. During the recruitment period (January 2024–February 2025), patients presenting for single-implant placement were screened consecutively against the eligibility criteria, and all eligible patients were approached for participation. Enrollment was restricted to eligible patients who provided written informed consent. Eligible sites were required to have undergone a minimum post-extraction healing period of 12 weeks, with clinical and radiographic evidence of adequate healing and sufficient bone volume for conventional implant placement without simultaneous bone augmentation. Each participant contributed one study implant and was required to be available for the scheduled 12-week follow-up. Restriction to healed sites was intended to minimize variability associated with immediate and early implant placement and differences in site-healing conditions that may influence longitudinal implant stability [15,16].

Participants were excluded in the presence of active untreated periodontitis, acute infection at the proposed implant site, uncontrolled systemic disease or conditions known to substantially affect bone metabolism or wound healing, current head-and-neck radiotherapy or chemotherapy, use of medications known to substantially interfere with bone metabolism, pregnancy, or the need for simultaneous bone augmentation/GBR [1,17,18]. Participants with a history of periodontitis were eligible when the disease had been treated and was clinically stable. Smoking and controlled diabetes were not exclusion criteria; instead, these exposures were prospectively documented and accounted for in the statistical analysis.

Sample-Size Calculation: Sample size was calculated prospectively using R version 4.3.0 for the primary global three-degree-of-freedom interaction between preoperative salivary aMMP-8 and time, based on implant stability quotient (ISQ) measurements obtained immediately after implant placement (T0) and at 4, 8, and 12 weeks. Based on published longitudinal data reporting ISQ standard deviations of approximately 8–9 units during early implant healing [19], the calculation assumed an ISQ standard deviation of 9 units, a compound-symmetric within-participant correlation of 0.50, a two-sided significance level of 0.05, and 80% power. The planning alternative specified differences in mean ISQ change from T0 of −1, −2, and −3 units at weeks 4, 8, and 12, respectively, per 1-standard-deviation increase in ln(aMMP-8), with no baseline exposure effect. Using a noncentral chi-square approximation for the global three-degree-of-freedom Wald test, 89 evaluable participants were estimated to provide approximately 80.3% power. To accommodate uncertainty in the assumed covariance structure, covariate adjustment, and incomplete follow-up, the recruitment target was increased to 125 participants. The sample-size calculation and its underlying effect-size assumptions were established before participant recruitment.

### 2.3. Preoperative Clinical and Periodontal Assessment

Before implant placement, participants underwent standardized medical, dental, and periodontal assessment. Age, sex, smoking status (never, former, or current), cumulative smoking exposure (pack-years), and HbA1c were recorded. A full-mouth periodontal examination was performed using a UNC-15 periodontal probe (Hu-Friedy, Chicago, IL, USA). Periodontal status was classified as periodontal health, gingivitis, or treated/stable periodontitis according to the 2017 World Workshop classification. Treated/stable periodontitis was defined by a documented history of periodontitis treated with periodontal therapy, with no evidence of ongoing periodontal progression, probing pocket depths ≤4 mm, no bleeding on probing at sites with probing depths ≥4 mm, and full-mouth bleeding on probing <10%. Periodontal stability was evaluated through clinical periodontal examination and available previous periodontal records [20]. Periodontal inflammatory burden was quantified as the percentage of sites exhibiting bleeding on probing (BOP%), and plaque accumulation as the percentage of examined sites/surfaces with detectable plaque.

### 2.4. Saliva Collection, Processing, Storage, and aMMP-8 Assay

Preoperative unstimulated whole saliva was collected between 09:00 and 11:00 after participants had refrained from eating, drinking (except water), smoking, and oral-hygiene procedures for at least 2 h. Following a 5 min rest period, saliva was collected by passive expectoration into sterile polypropylene tubes over 5 min. Specimens were immediately placed on ice, centrifuged at 1500× *g* for 15 min at 4 °C, aliquoted, and stored at −80 °C until analysis (median storage duration, 4.0 months; interquartile range [IQR], 2.5–5.8 months). Each specimen underwent one freeze–thaw cycle.

Salivary active matrix metalloproteinase-8 (aMMP-8) was quantified using the QuickZyme Human MMP-8 Activity Assay (QuickZyme Biosciences, Leiden, The Netherlands; catalog no. QZBmmp8H), an antibody-capture, activity-based colorimetric assay. Endogenous active MMP-8 was measured without 4-aminophenylmercuric acetate (APMA)-mediated activation of latent pro-MMP-8 in the saliva specimens; calibration standards were prepared according to the manufacturer’s activation protocol. Specimens were initially diluted 1:100 and analyzed in duplicate using the manufacturer’s 2 h protocol. Absorbance was measured at 405 nm, and concentrations were derived from plate-specific calibration curves and corrected for the final corresponding sample dilution factor and expressed as ng/mL of original saliva. Laboratory personnel were blinded to subsequent implant stability quotient (ISQ) measurements.

The manufacturer-reported limit of detection (LOD) was 0.10 ng/mL, and the reported lower and upper limits of quantification (LLOQ and ULOQ) were 0.19 and 12.0 ng/mL, respectively. At the initial 1:100 dilution, these corresponded to an effective detection limit of 10 ng/mL and a quantification range of 19–1200 ng/mL in original saliva.

A total of 125 specimens were analyzed across four analytical plates and two batches. Specimens were randomly allocated across plates and batches, with allocation balanced by periodontal status. Plate-specific specimen counts were 31, 31, 31, and 32, respectively. Duplicate measurements were accepted according to a laboratory coefficient of variation (CV) threshold of ≤10%. Four specimens (3.2%) initially exceeded this threshold and were successfully reanalyzed, with no exclusions due to analytical imprecision. The median duplicate CV was 3.8% (IQR, 2.6–5.4%). Study-specific intra-assay and inter-assay precision were evaluated using replicate measurements within runs and repeated measurements of the same quality-control material across independent runs, yielding CVs of 4.2% and 7.6%, respectively.

Calibration curves were generated independently for each plate, with R^2^ values of 0.996, 0.997, 0.998, and 0.997 (mean, 0.997). Spike recovery was evaluated in six independent saliva matrices at three concentration levels in triplicate (94–106%), while dilution recovery was assessed in six specimens at 1:100, 1:200, and 1:400, where quantifiable (95–105%).

Three specimens (2.4%) initially exceeded the upper calibration limit at 1:100 dilution and were successfully reanalyzed at 1:200 dilution (effective quantification range, 38–2400 ng/mL in original saliva). Their dilution-corrected concentrations were included after confirmation that the repeat measurements fell within the reported quantification range. No specimens were below the applicable LLOQ, and none were excluded for analytical reasons.

### 2.5. Implant Placement and Standardization of Surgical Procedures

All procedures were performed under local anesthesia using a standardized surgical protocol by two experienced implant clinicians, each with at least five years of experience in implant surgery. Both clinicians followed the same surgical protocol to minimize interoperator variability. Following full-thickness mucoperiosteal flap elevation, osteotomies were prepared under sterile saline irrigation according to the manufacturer’s drilling protocol. The drilling sequence was adapted for sites with low-density bone, with the final drill diameter selected according to the planned implant diameter, intraoperatively assessed bone quality, and the applicable manufacturer’s recommendations. A single-implant system (IS-II Active, NeoBiotech, Seoul, Republic of Korea), featuring a sandblasted and acid-etched (S.L.A.) titanium surface produced by hydroxyapatite-particle blasting followed by dual acid etching, was used throughout the study. Implants were placed in healed edentulous sites according to prosthetically driven planning. Implant site, jaw, length, and diameter were recorded. Anatomical implant location was classified as anterior (incisors and canines) or posterior (premolars and molars) based on the recorded Fédération Dentaire Internationale (FDI) tooth-position codes. Bone quality was assessed intraoperatively according to the Lekholm–Zarb classification (D1–D4), and final insertion torque was recorded in N·cm.

A standardized non-submerged, transmucosal healing protocol was used. Healing abutments were connected at implant placement, and implants remained free from functional loading throughout the 12-week observation period. This permitted serial RFA without surgical re-entry. Sites requiring simultaneous bone augmentation were excluded, and all participants received standardized postoperative care and instructions. In patients without contraindications, postoperative analgesia consisted of ibuprofen (400 mg orally every 6–8 h as needed) during the first 48 h. Participants were instructed to rinse with 0.12% chlorhexidine gluconate twice daily for 7 days, maintain oral hygiene while avoiding direct mechanical brushing of the surgical wound during initial healing, follow a soft diet, avoid direct mechanical loading at the implant site, and refrain from smoking during healing. They were also instructed to report persistent bleeding, increasing pain, swelling, or other signs of postoperative complications. A postoperative review was scheduled approximately 7 days after implant placement to assess soft-tissue healing and remove nonresorbable sutures when applicable. Subsequent clinical evaluations and RFA measurements were scheduled at 4, 8, and 12 weeks after implant placement.

### 2.6. Implant Stability Assessment and Follow-Up

Implant stability was assessed by resonance frequency analysis (RFA) using an Osstell ISQ device (Osstell AB, Gothenburg, Sweden) with a SmartPeg Type 05 (reference 100376) immediately after implant placement (T0) and at 4, 8, and 12 weeks. The SmartPeg was attached directly to the implant using the manufacturer’s SmartPeg Mount and secured by manual finger-tightening in accordance with the manufacturer’s instructions, without the use of a torque-measuring instrument. The measurement probe was positioned close to the SmartPeg without contact, following the manufacturer’s recommended distance and angulation. The same standardized measurement protocol was applied at all assessment visits.

RFA measurements were performed by two examiners, both blinded to preoperative salivary active matrix metalloproteinase-8 (aMMP-8) results. Each participant was assigned to one examiner who performed the baseline and subsequent assessments whenever feasible to minimize within-participant examiner variability. Before study assessments, both examiners underwent standardized training in Osstell device operation, SmartPeg attachment and tightening, probe positioning, anatomical measurement orientation, and ISQ recording. Formal inter-examiner calibration was conducted using 20 implants. Inter-examiner reliability was assessed using a two-way random-effects, absolute-agreement, single-measure intraclass correlation coefficient [ICC (2,1)], demonstrating good agreement between examiners (ICC = 0.89; 95% confidence interval [CI]: 0.86–0.915).

At each assessment, two implant stability quotient (ISQ) measurements were obtained in approximately perpendicular buccolingual and mesiodistal directions, and their arithmetic mean was used for analysis. Inter-directional agreement was assessed using a pooled absolute-agreement intraclass correlation coefficient (ICC = 0.91; 95% confidence interval [CI]: 0.88–0.93), supplemented by visit-specific ICC estimates and Bland–Altman analysis of the mean difference and 95% limits of agreement. Because the readings represented different anatomical orientations, inter-directional agreement was not interpreted as intra-examiner test–retest repeatability. Same-direction test–retest repeatability was not formally assessed. Direction-specific analyses were performed to examine whether the primary longitudinal findings were sensitive to measurement orientation. At follow-up visits, the healing abutment was temporarily removed for SmartPeg attachment and reconnected after measurement.

Early implant failure was defined as implant removal or loss before completion of the 12-week follow-up because of failure to achieve or maintain clinical stability. Time and reason for failure were recorded. ISQ measurements scheduled after implant failure were considered structurally unavailable because no implant remained available for measurement. These observations were distinguished from intermittently missing measurements in participants whose implants remained in situ. The potential influence of implant failure on the longitudinal findings was examined through separate failure-inclusive sensitivity analyses, as described in Section 2.8.

### 2.7. Outcomes, Exposure, Covariates, and Bias Control

The primary exposure was preoperative salivary aMMP-8 concentration. The primary outcome was the longitudinal trajectory of mean ISQ across T0, 4, 8, and 12 weeks. The primary estimands were the adjusted differences in mean ISQ change from T0 at weeks 4, 8, and 12 per 1-SD higher preoperative ln(aMMP-8). The study was designed to investigate the association between preoperative salivary aMMP-8 and longitudinal implant stability. Although the study was conducted prospectively, no publicly registered protocol or independently verifiable dated statistical analysis plan was available to establish the timing of the detailed analytical specifications relative to outcome-data access. Early implant failure through 12 weeks was a secondary descriptive outcome. Covariates included age, sex, smoking status, diabetes status, periodontal status, unstimulated salivary flow, jaw, implant length, implant diameter, insertion torque, and bone quality. Insertion torque and bone quality were additionally modeled in interaction with time. HbA1c was evaluated separately as a continuous measure of glycemic control in sensitivity analysis S4f, replacing diabetes status. Standardized saliva collection and laboratory procedures, use of a single-implant system, standardized RFA assessment, one implant per participant, prospective ascertainment of covariates, and blinding of laboratory personnel to ISQ measurements were used to reduce measurement and confounding bias.

### 2.8. Statistical Analysis

Statistical analyses were performed using R version 4.3.3 (mmrm 0.3.19, emmeans 1.10.0, mice 3.16.0, rms 6.7.1, logistf 1.26.1, and irr 0.85). All tests were two-sided, with a significance level of 0.05. Continuous variables were summarized as mean ± standard deviation (SD) or median [interquartile range], and categorical variables as frequencies and percentages. Salivary aMMP-8 concentrations were natural-log transformed and standardized, with effect estimates expressed per 1-SD increase in ln(aMMP-8). All baseline covariates were complete for the 125 participants; therefore, no covariate imputation was required.

For the primary analysis, implant stability quotient (ISQ) measurements obtained immediately after placement (T0) and at 4, 8, and 12 weeks were analyzed using a mixed model for repeated measures (MMRM). Time was modeled categorically, with an unstructured within-participant covariance matrix, restricted maximum likelihood estimation, and Kenward–Roger inference. Fixed effects included standardized ln(aMMP-8), time, and their interaction, adjusted for age, sex, smoking status, diabetes status, periodontal status, salivary flow, jaw, implant length and diameter, insertion torque, and bone quality (D1–D2 versus D3–D4). Interactions of insertion torque and bone quality with time were included to account for their potentially time-varying associations with implant stability. These variables were treated as prognostic covariates reflecting site characteristics and primary mechanical stability rather than presumed mediators of the aMMP-8 association. Their influence was examined through alternative adjustment specifications. Continuous covariates were modeled linearly. Alternative functional forms were examined using restricted cubic splines or categorical coding, as appropriate, and compared using likelihood-ratio tests. Baseline ISQ was incorporated as part of the repeated outcome rather than as a separate covariate. The primary estimands were the adjusted differences in ISQ change from T0 at each follow-up visit per 1-SD higher ln(aMMP-8). The global aMMP-8 × time interaction was evaluated using a three-degree-of-freedom test, with week-specific contrasts reported as estimates, 95% confidence intervals (CIs), and Holm-adjusted *p* values.

Model assessment and sensitivity analyses. Model assumptions were evaluated using residual diagnostics, alternative continuous-covariate specifications, generalized variance inflation factors, and condition indices. Additional sensitivity analyses were conducted to assess the robustness of the primary findings and examine alternative modeling assumptions. These included baseline constraints (S1), baseline-adjusted postplacement ISQ (S2), alternative exposure scales and nonlinear associations (S3), covariate specifications (S4), survivor restriction (S5), complete-case analysis (S6), multiple imputation (S7), influential observations (S8), and alternative covariance structures (S9). Exposure nonlinearity was assessed using restricted cubic splines with knots at the 10th, 50th, and 90th percentiles of ln(aMMP-8). Additional sensitivity analyses were conducted to assess the robustness of the primary findings and examine alternative modeling assumptions.

Missing data and implant failure. The primary analysis estimated ISQ trajectories while implants remained in situ. This while-in situ estimand describes the association between preoperative aMMP-8 and longitudinal ISQ while implants remained present and measurable; it does not estimate an ISQ trajectory after implant failure or the association with implant survival. Intermittently missing ISQ measurements were accommodated through likelihood-based estimation under a missing-at-random assumption, whereas post-failure measurements were considered structurally unavailable. Multiple imputation of intermittently missing observations was performed using chained equations with predictive mean matching (five donors, 50 imputations, and 20 iterations), followed by Rubin’s pooling with small-sample adjustments (S7). Failure-inclusive sensitivity analyses evaluated unfavorable ISQ composites (S10), hypothetical post-failure imputation with delta-adjusted tipping-point analyses (S11), and a worst-rank composite analyzed using proportional-odds regression (S12). These supplementary analyses were conducted to assess robustness to alternative assumptions regarding implant failure and were distinguished from the primary estimand. Implant failure frequency was summarized using an exact Clopper–Pearson 95% CI. Failed and surviving implants were compared descriptively, with exploratory unadjusted Firth logistic regression owing to the limited number of failures.

Periodontal confounding. Differences in aMMP-8 concentrations across periodontal categories were assessed using Kruskal–Wallis tests followed by Holm-adjusted Dunn comparisons, with geometric mean ratios estimated using log-linear regression. Associations with bleeding on probing (BOP%) and plaque percentage were evaluated using Spearman and partial rank correlations. Sensitivity to measured periodontal inflammation was examined by additionally adjusting the MMRM for BOP%, plaque percentage, or both, and by restricting analyses to periodontally healthy participants. Potential effect modification was assessed using the aMMP-8 × time × periodontal status interaction. Subgroup analyses were considered exploratory.

Measurement agreement. Agreement between buccolingual and mesiodistal ISQ measurements was evaluated using absolute-agreement intraclass correlation coefficients, Bland–Altman analysis, within-participant SD, and an inter-directional agreement coefficient calculated as 2.77 times the within-participant SD. Direction-specific MMRMs were fitted to assess the influence of measurement orientation on the longitudinal associations. Inter-directional agreement was distinguished from same-direction test–retest repeatability.

## 3. Results

### 3.1. Study Population and Participant Characteristics

**Figure 1:**
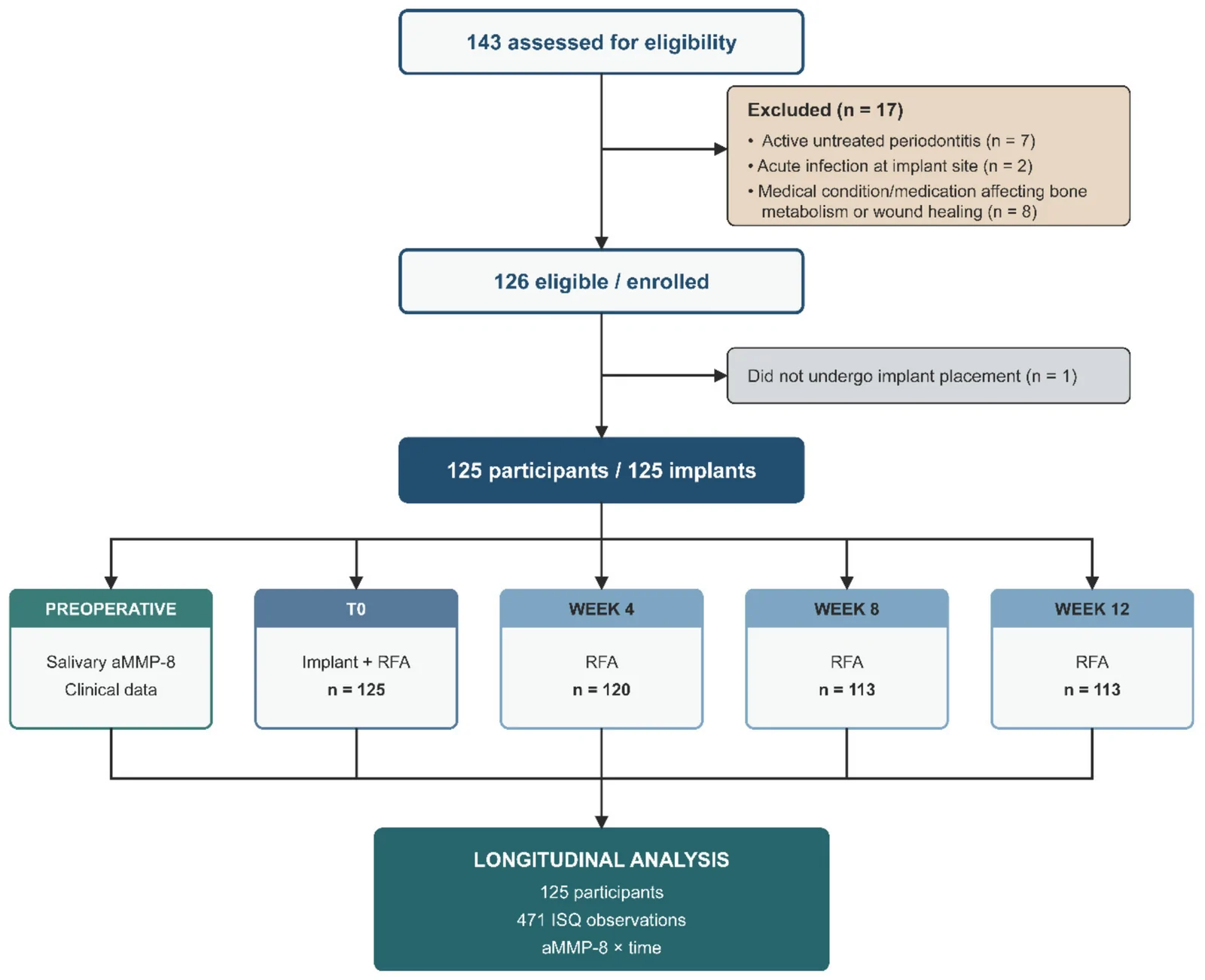
Study design, participant flow and longitudinal assessment schedule.

A total of 143 patients were assessed for eligibility; 17 were excluded and one eligible participant did not undergo implant placement, resulting in 125 participants receiving 125 study implants (Figure 1). All 125 participants contributed a T0 observation. Mean age was 56.4 ± 10.0 years, and 67 (53.6%) were women. Most participants had never smoked (69, 55.2%), 12 (9.6%) had diabetes, and 49 (39.2%) had treated/stable periodontitis. Median preoperative salivary aMMP-8 was 142.3 ng/mL [IQR, 95.9–223.8], and median salivary flow was 0.39 mL/min [IQR, 0.31–0.49]. Sixty-five implants (52.0%) were placed in the maxilla, and 106 (84.8%) were located in posterior sites. Seventy-four sites (59.2%) had D3 + D4 bone quality, and mean insertion torque was 36.3 ± 9.3 N·cm (Table 1).

### 3.2. Observed Early Implant-Stability Trajectory

**Figure 2:**
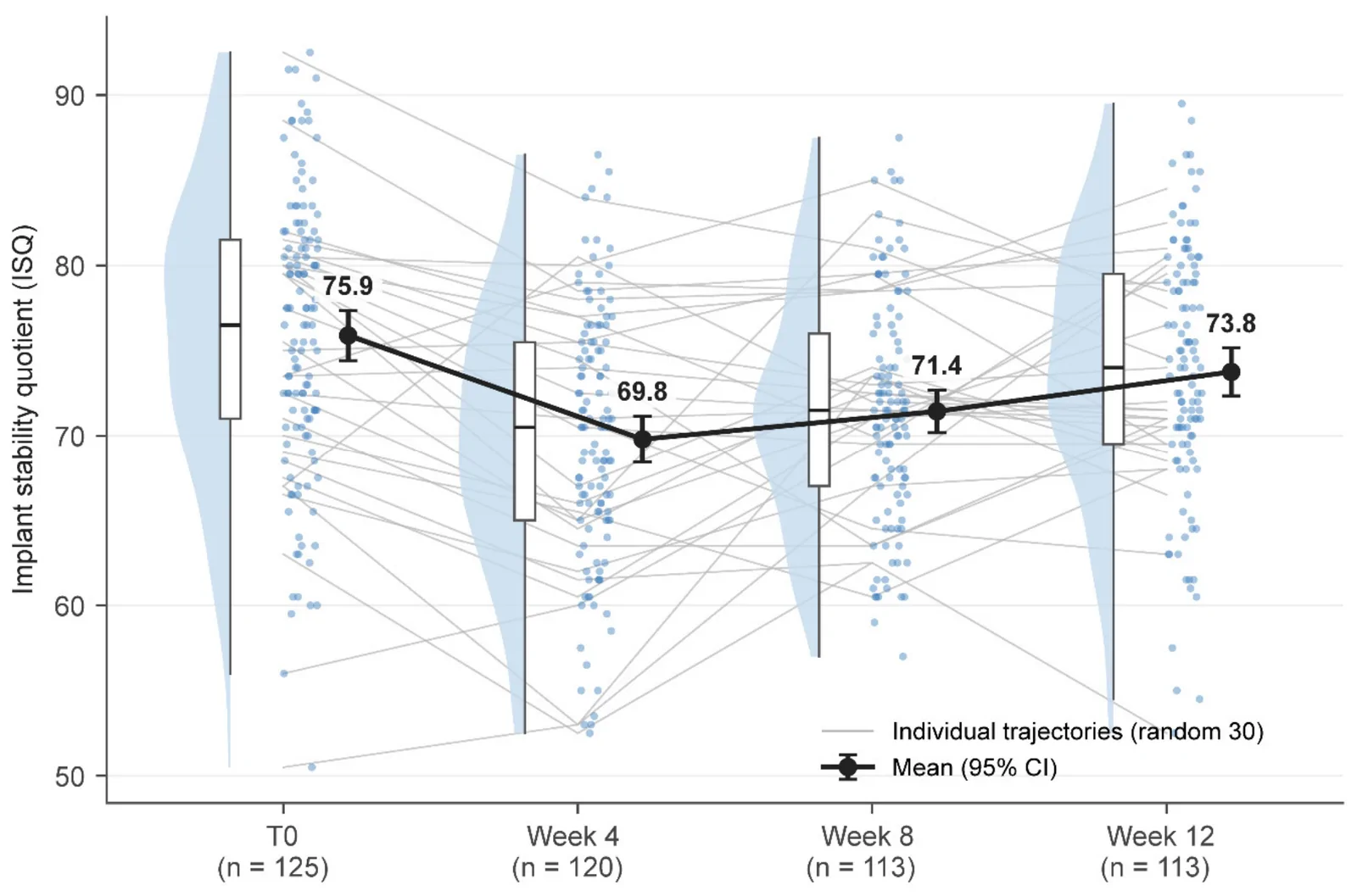
Observed ISQ trajectory during the 12-week follow-up. Half-violin plots show the distribution of ISQ at each visit, with box plots showing the median and interquartile range and individual observations (dots). Grey lines show individual trajectories for a random subset of 30 participants. The black line shows the observed visit-specific mean and 95% CI. *n* denotes the number of observed measurements at each visit. Values are unadjusted; the figure does not depict the adjusted association between preoperative aMMP-8 and ISQ trajectory.

Of 500 scheduled ISQ measurements, 471 (94.2%) were observed, 16 (3.2%) were ordinarily missing in 13 participants, and 13 (2.6%) were structurally unavailable after implant failure (Table 2). One hundred and six participants (84.8%) had complete ISQ data at all four visits. Observed ISQ decreased after implant placement and subsequently increased during follow-up (Figure 2). Mean ISQ was 75.9 ± 8.3 at placement, decreased to 69.8 ± 7.5 at week 4 (mean within-participant change, −6.05 ± 5.82), and increased at week 8 (71.4 ± 6.7) and week 12 (73.8 ± 7.6). At week 12, mean ISQ remained 2.21 points below T0 (mean within-participant change, −2.21 ± 7.98). These unadjusted values describe the observed ISQ trajectory; the adjusted association with preoperative aMMP-8 is reported below.

### 3.3. Primary Association: aMMP-8 and ISQ Trajectory

**Figure 3:**
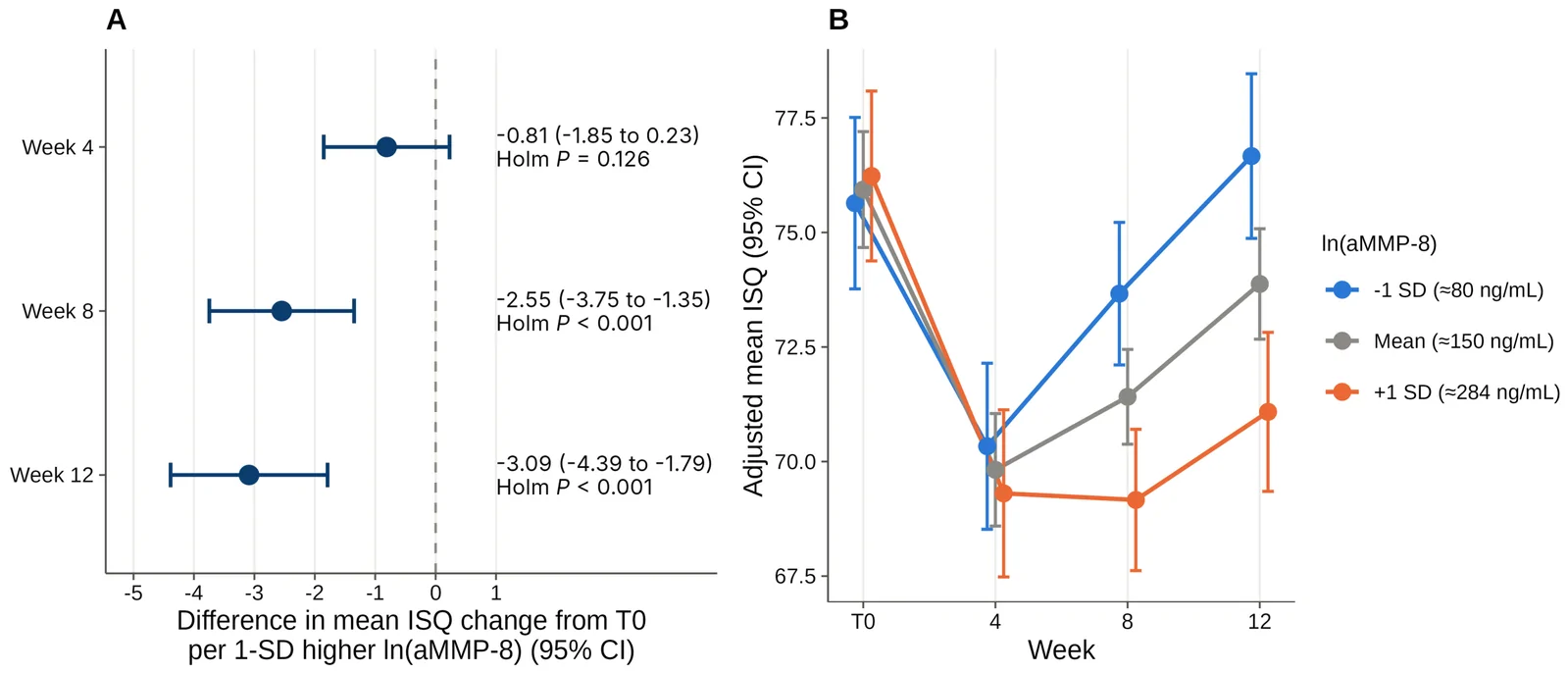
Association between preoperative salivary aMMP-8 and early implant-stability trajectory. (**A**) Time-specific adjusted differences in mean ISQ change from T0 per 1-SD higher preoperative ln(aMMP-8) at weeks 4, 8, and 12. Points represent estimates and horizontal bars represent 95% CIs; the dashed vertical line denotes no difference. Holm-adjusted *p* values are shown. (**B**) Model-estimated mean ISQ trajectories with 95% CIs at −1 SD, the mean, and +1 SD of preoperative ln(aMMP-8), corresponding to approximately 80, 150, and 284 ng/mL, respectively. Marginal means were estimated from the primary MMRM and averaged over the observed covariate distribution. The global aMMP-8 × time interaction was F(3, 112.1) = 8.81, *p* < 0.001.

The primary analysis included all 125 participants and 471 observed ISQ measurements. The global aMMP-8 × time interaction was statistically significant (F(3, 112.1) = 8.81; *p* < 0.001), indicating that the 12-week ISQ trajectory differed according to preoperative aMMP-8 (Table 3; Table S1, panel A). In the gated week-specific contrasts, each 1-SD higher preoperative ln(aMMP-8) was not clearly associated with ISQ change from T0 to week 4 (−0.81 ISQ units; 95% CI, −1.85 to 0.23; Holm-adjusted *p* = 0.126), but was associated with a less favorable change in ISQ from T0 at week 8 (−2.55 ISQ units; 95% CI, −3.75 to −1.35; Holm-adjusted *p* < 0.001) and week 12 (−3.09 ISQ units; 95% CI, −4.39 to −1.79; Holm-adjusted *p* < 0.001) (Table 3; Figure 3A). The adjusted cross-sectional association between aMMP-8 and ISQ at T0 was close to null (0.30 ISQ units per 1-SD higher ln(aMMP-8); 95% CI, −1.07 to 1.66; *p* = 0.669; Table S1, panel A). Full fixed-effect estimates, the estimated covariance matrix, collinearity diagnostics, and functional-form assessments are reported in Table S1, panels A–D, respectively.

Model-estimated trajectories were consistent with the time-specific contrasts (Figure 3B). Adjusted mean ISQ at T0 was similar at −1 SD, the mean, and +1 SD of ln(aMMP-8) (approximately 80, 150, and 284 ng/mL: 75.6, 75.9, and 76.2, respectively), with little separation at week 4. Greater separation was observed at weeks 8 and 12, with lower adjusted mean ISQ at higher preoperative aMMP-8 levels. At week 12, adjusted mean ISQ was 76.7 (95% CI, 74.9–78.5), 73.9 (72.7–75.1), and 71.1 (69.3–72.8) at −1 SD, the mean, and +1 SD of ln(aMMP-8), respectively.

### 3.4. Robustness and Sensitivity Analyses

**Figure 4:**
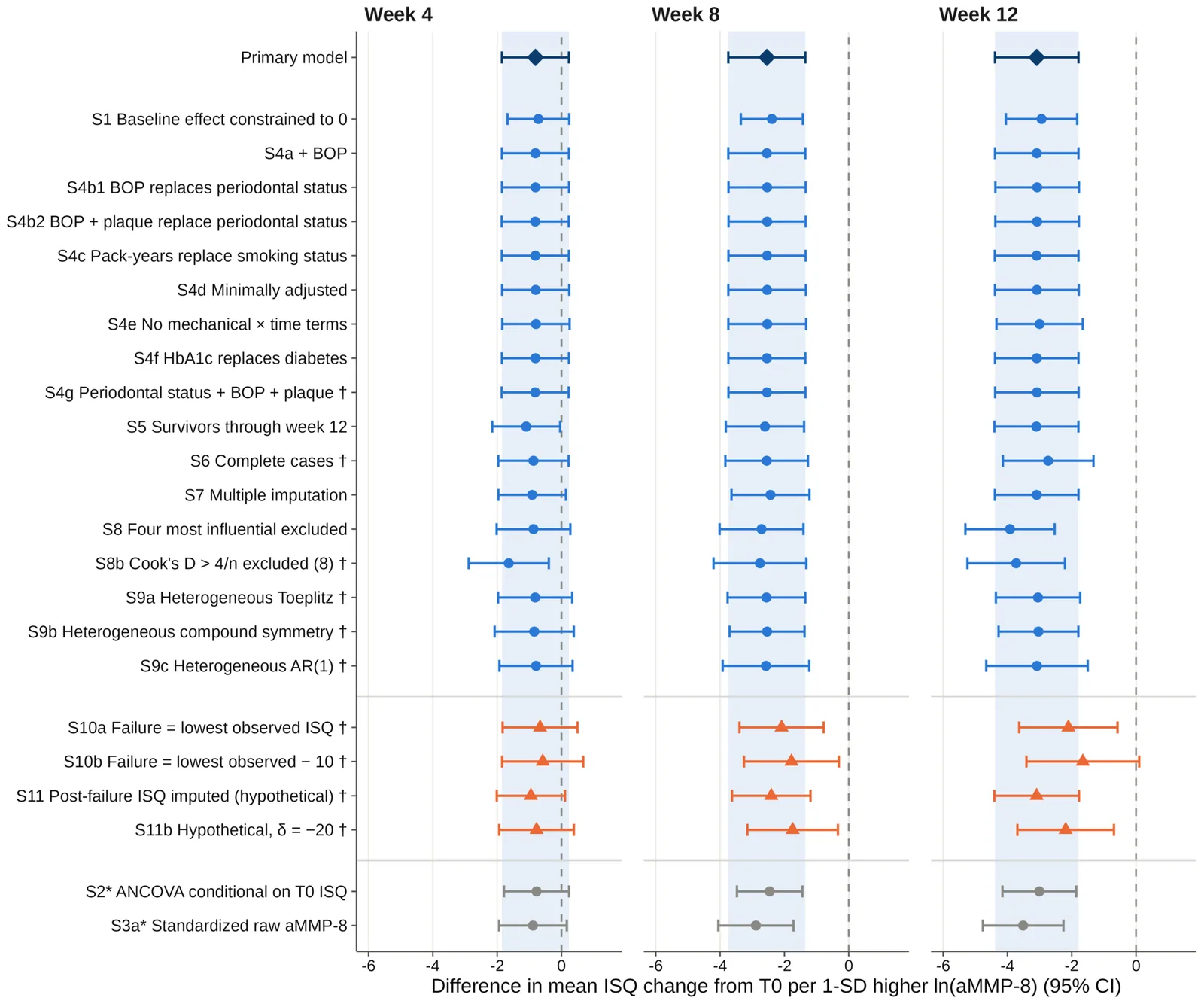
Robustness of the time-specific associations between preoperative aMMP-8 and ISQ trajectory. Panels show estimates at week 4 (A), week 8 (B), and week 12 (C). Points and horizontal bars represent adjusted differences in mean ISQ change from T0 with 95% CIs per 1-SD higher preoperative ln(aMMP-8). The diamond denotes the primary estimate, the shaded band its 95% CI, and the dashed vertical line denotes no difference. Analyses below the divider (*) use either a different estimand (S2, postbaseline ISQ conditional on T0 ISQ) or a different exposure scale (S3a, per 1-SD of untransformed aMMP-8 [132.7 ng/mL]) and are therefore not directly comparable in magnitude with the primary estimate. † Post hoc sensitivity analyses. The restricted cubic spline analysis (S3b) is presented separately in Figure S1.

The primary findings were generally consistent across conventional sensitivity analyses examining alternative model specifications, covariate adjustments, missing-data assumptions, and analytic populations (Figure 4; Table S2). The global aMMP-8 × time interaction remained statistically significant in such analyses, and the week-8 and week-12 estimates remained negative with 95% CIs excluding zero. Results were materially unchanged after constraining the baseline exposure effect to zero (S1) and across alternative covariate specifications (S4a–S4g), complete-case analysis (S6), and alternative covariance structures (S9). Multiple imputation of the 16 ordinarily missing observations (S7; 50 imputations) yielded similar estimates at week 8 (−2.44; 95% CI, −3.65 to −1.22) and week 12 (−3.09; 95% CI, −4.39 to −1.79; global F(3, 112.9) = 8.28, *p* < 0.001). The postbaseline ANCOVA conditional on T0 ISQ (S2) and the standardized raw-aMMP-8 model (S3a), which target a different estimand and use a different exposure scale, respectively, were directionally consistent with the primary analysis but are not directly comparable in effect magnitude.

Restriction to the 119 implants surviving through 12 weeks (S5) yielded similar estimates at week 8 (−2.61; 95% CI, −3.82 to −1.39) and week 12 (−3.10; 95% CI, −4.40 to −1.79); the week-4 estimate was −1.10 (95% CI, −2.15 to −0.04; *p* = 0.042). The four participants with the largest generalized Cook’s distances were excluded in S8 (121 participants, 455 observations), and their exclusion did not attenuate the associations at week 8 (−2.71; 95% CI, −4.02 to −1.41) or week 12 (−3.92; 95% CI, −5.31 to −2.53). The week-4 95% CI also excluded zero in S8b, which excluded eight participants exceeding the conventional 4/n Cook’s distance threshold; in the remaining conventional sensitivity analyses, the week-4 95% CIs included zero (Table S2). The restricted cubic spline analysis (S3b) suggested departure from a linear exposure–response relationship on the ln(aMMP-8) scale (*p* = 0.028). Accordingly, the primary linear-model estimates should be interpreted as summary associations under the assumed log-linear specification rather than constant effects across the entire biomarker distribution (Supplementary Table S1, panel D and Figure S1). The spline analysis also provided evidence of departure from log-linearity (F(3, 110.8) = 3.15, *p* = 0.028; Supplementary Table S1). Although the direction and timing of the association were broadly consistent across exposure specifications, the evidence of nonlinearity suggests that the primary linear-model estimates should be interpreted as model-based summaries rather than as uniform effects across the entire aMMP-8 concentration range.

### 3.5. Periodontal Inflammatory Burden and aMMP-8

Preoperative salivary aMMP-8 concentrations differed significantly across periodontal categories (Kruskal–Wallis χ^2^(2) = 19.98, *p* < 0.001), with median concentrations of 106.2, 184.2, and 158.8 ng/mL in participants with periodontal health, gingivitis, and treated/stable periodontitis, respectively (Table 4). Salivary aMMP-8 was positively correlated with bleeding on probing (Spearman’s ρ = 0.47) and plaque percentage (ρ = 0.36; both *p* < 0.001). Additional correlation analyses are reported in Table S3, panel A. Additional adjustment for these inflammatory parameters did not materially change the primary week-12 association (−3.08 ISQ units; 95% CI, −4.38 to −1.78; Table S2). In the periodontal-health subgroup (*n* = 43), the week-12 association remained negative but was not statistically significant (−2.12; 95% CI, −5.00 to 0.76), and the global aMMP-8 × time interaction was not significant (*p* = 0.389). However, formal interaction testing did not provide evidence that the association differed by periodontal status (*p* = 0.269; Table S3).

### 3.6. Implant Failure

Six of 125 implants failed during the 12-week follow-up (4.8%; exact 95% Clopper–Pearson CI, 1.8–10.2%). Median time to failure was 5.25 weeks (range, 2.8–11.5 weeks). Four failures were attributed to failure of osseointegration, and two to early mobility/loss of stability. All six failures occurred in participants classified as periodontally healthy. Compared with surviving implants, failed implants had lower preoperative aMMP-8 (median, 105.3 vs. 151.5 ng/mL), insertion torque (27.9 vs. 36.6 N·cm), and T0 ISQ (70.0 vs. 77.5). Given the small number of events, these comparisons are descriptive and should not be interpreted as evidence of independent predictors of failure (Table S4).

Failure-inclusive sensitivity analyses were conducted to evaluate the influence of alternative assumptions regarding unobserved post-failure ISQ outcomes. When post-failure observations were assigned the lowest observed visit-specific ISQ, the week-12 association remained negative (−2.10 ISQ units; 95% CI, −3.63 to −0.58; global interaction *p* = 0.011). However, applying an additional 10-unit penalty attenuated the week-12 estimate (−1.66; 95% CI, −3.41 to 0.10), and the global interaction was no longer statistically significant (*p* = 0.112). Hypothetical post-failure imputation with a −25-unit delta adjustment also yielded a nonsignificant global interaction. The worst-rank composite analysis, which evaluated a different outcome scale, yielded a week-12 odds ratio of 0.51 (95% CI, 0.35–0.74). The assigned post-failure ISQ values were hypothetical rather than observed measurements. The statistical significance of the association was sensitive to the imposed penalties, and the resulting estimates, including those from the worst-rank composite analysis, should not be interpreted as directly interchangeable with the primary MMRM estimate (Table S5; Figure S2).

## 4. Discussion

The principal finding of this prospective cohort study was that preoperative salivary aMMP-8 was associated with the subsequent trajectory of dental implant stability during the first 12 weeks of healing. This association was time-dependent rather than evident at implant placement: preoperative aMMP-8 showed little evidence of an association with ISQ at T0, and no clear differential change was observed at week 4, whereas higher aMMP-8 levels were associated with progressively less favorable changes in ISQ at weeks 8 and 12. Importantly, this temporal pattern remained consistent across conventional sensitivity analyses examining addressing alternative baseline constraints, exposure parameterization, periodontal and metabolic covariate specifications, missing-data assumptions, and influential observations, although failure-inclusive analyses demonstrated attenuation under some assumptions concerning post-failure outcomes. Taken together, these findings indicate that the association between preoperative aMMP-8 and implant stability emerged during the healing period rather than reflecting differences in primary implant stability at placement.

The timing of the observed association is noteworthy in the context of the transition from primary to secondary implant stability. At placement, implant stability is predominantly influenced by mechanical engagement with the surrounding bone and by factors such as local bone characteristics, implant geometry, and surgical preparation [2]. During early healing, primary mechanical stability may change as peri-implant bone formation and remodeling progressively contribute to secondary stability, producing a dynamic stability pattern over time [2,4,6]. The near-null association between preoperative aMMP-8 and T0 ISQ in the present study therefore suggests that the subsequent trajectory differences were not simply attributable to lower primary mechanical stability among participants with higher biomarker levels. Notably, the absence of a clear differential change at week 4, followed by increasingly negative differences at weeks 8 and 12, is compatible with the possibility that interindividual variation in the preoperative host-response state becomes more relevant as healing progresses. This temporal pattern is biologically plausible but should not be interpreted as evidence that elevated salivary aMMP-8 directly impaired osseointegration, because RFA characterizes the mechanical stability of the implant–tissue complex and does not directly measure the underlying cellular or histological processes [2].

A biological interpretation of these findings should consider what preoperative salivary aMMP-8 represents. MMP-8 is a predominantly neutrophil-derived collagenase involved in extracellular-matrix turnover, and conversion from its latent form to enzymatically active MMP-8 is particularly relevant to ongoing collagenolytic activity in inflammatory periodontal tissues [7,9]. Consistent with this role, elevated aMMP-8 concentrations in oral fluids have been associated with periodontal inflammatory burden and tissue breakdown, while peri-implant sulcular-fluid aMMP-8 has shown diagnostic value in established peri-implantitis [7,10]. Accordingly, the preoperative salivary measurement in the present study may reflect interindividual variation in the pre-existing oral host-response and tissue-remodeling milieu before the surgical insult. Such variation could plausibly be relevant during subsequent healing, when inflammatory regulation, extracellular-matrix turnover, and bone remodeling contribute to the evolving implant–tissue interface. Nevertheless, salivary aMMP-8 is a mouth-level biomarker and cannot localize molecular activity to the future implant site. It should therefore be interpreted here as a marker of the preoperative host-response milieu associated with the subsequent stability trajectory, rather than as evidence of a direct aMMP-8-mediated effect at the bone–implant interface.

The present findings extend two related but distinct areas of previous implant research. First, studies of MMP-8 and aMMP-8 in implant dentistry have largely focused on peri-implant sulcular or crevicular fluid obtained around existing implants, primarily to distinguish peri-implant health from mucositis or peri-implantitis or to monitor peri-implant inflammatory responses [10,14]. Recent systematic evidence supports higher MMP-8/aMMP-8 levels in peri-implantitis than in healthy peri-implant conditions, while also highlighting heterogeneity in measurement approaches and the need for further prospective validation [10,21]. Second, prospective studies have shown that biological markers can be examined alongside serial changes in implant stability during healing. Tirachaimongkol et al. (2016), for example, repeatedly assessed ISQ together with peri-implant osteocalcin and alkaline phosphatase over 12 weeks and reported weak associations between these local bone-turnover markers and implant stability [12]. However, this approach evaluated biomarkers sampled locally during the healing period rather than a host-response marker obtained before implant placement. To our knowledge, the present study is the first prospective clinical investigation to examine the association between preoperative salivary aMMP-8 and the subsequent longitudinal trajectory of implant stability during early healing. By establishing biomarker status before surgery and repeatedly assessing ISQ through 12 weeks, the present study extends previous peri-implant biomarker research from contemporaneous assessment of established peri-implant conditions toward investigation of whether the preoperative host-response profile is associated with subsequent variation in early implant stability.

The potential clinical relevance of these findings lies in the availability of salivary aMMP-8 before implant placement, when information regarding the subsequent stability trajectory is not yet available. Unlike insertion torque and T0 ISQ, which characterize mechanical conditions at the time of implant placement, a preoperative salivary biomarker may provide complementary information on the host-response milieu preceding surgery. The observation that aMMP-8 showed little association with T0 ISQ but was associated with subsequent changes at weeks 8 and 12 after adjustment for conventional clinical and mechanical factors supports the possibility that its information is not simply redundant with primary mechanical stability. Nevertheless, the adjusted differences of −2.55 and −3.09 ISQ units at weeks 8 and 12, respectively, indicate statistically lower stability trajectories rather than established clinically meaningful impairment of healing. These modest differences may reflect variation in the transition from primary mechanical stability to biologically mediated secondary stability; however, they do not establish delayed osseointegration, increased implant failure risk, or a need to modify loading decisions. No universally accepted minimally important ISQ difference is available for this setting. Although inter-directional measurement agreement was high, same-direction test–retest repeatability was not assessed; therefore, it remains uncertain whether differences in this magnitude exceed measurement variability or warrant changes in clinical management.

Moreover, the present study evaluated longitudinal association rather than prognostic performance and did not assess discrimination, calibration, clinically actionable thresholds, or incremental predictive value beyond established clinical and mechanical factors. Thus, an association that persists after covariate adjustment should not be interpreted as evidence that adding aMMP-8 improves clinical prediction. Accordingly, these findings should not yet be interpreted as validating salivary aMMP-8 for preoperative risk stratification or for guiding loading or follow-up protocols. Rather, they provide a rationale for external prospective validation and direct comparison of prediction models with and without aMMP-8 to determine whether preoperative aMMP-8 adds clinically useful prognostic information to conventional implant assessment and, ultimately, whether biomarker-informed monitoring improves patient outcomes.

This study has several strengths, including its prospective design, measurement of salivary aMMP-8 before implant placement, and repeated assessment of implant stability at prespecified intervals, which established a clear temporal sequence between biomarker measurement and the subsequent stability trajectory. The one-participant–one-implant design avoided additional within-participant clustering, while the longitudinal analysis incorporated all available ISQ measurements while implants remained in situ and accounted for relevant demographic, periodontal, salivary, implant-, site-, and mechanically related covariates. The principal associations were generally consistent across alternative model specifications, exposure parameterizations, covariate definitions, and influence analyses. However, the magnitude and statistical significance of the associations were sensitive to certain failure-inclusive assumptions, particularly at week 12, limiting claims of robustness across all plausible post-failure scenarios.

Several limitations warrant consideration. First, the observational design precludes causal inference, and residual confounding, particularly from subclinical periodontal inflammation, remains possible despite multivariable adjustment. Although the association was directionally consistent among periodontally healthy participants (*n* = 43), the wide confidence interval limits subgroup-specific conclusions. Second, the single-center setting, standardized implant system, and cohort characteristics may limit generalizability. Third, a single preoperative whole-saliva aMMP-8 measurement cannot localize inflammation to the implant site or capture postoperative biomarker changes. Fourth, RFA measures mechanical implant stability rather than histological osseointegration. Fifth, the 12-week follow-up precludes conclusions regarding long-term stability, peri-implant disease, or implant survival. Sixth, the absence of an independently verifiable dated statistical analysis plan limits confirmation that all analytical specifications were finalized before outcome assessment. Seventh, exploratory spline analysis suggested departure from log-linearity (*p* = 0.028), indicating that the association may vary across aMMP-8 concentrations. Thus, the primary linear estimates may not apply uniformly across the biomarker range.

Finally, only six early implant failures occurred, precluding reliable failure-risk modeling. Potential informative truncation may have influenced later ISQ associations, and survivor-only analyses cannot resolve this concern. Failure-inclusive sensitivity analyses depended on hypothetical post-failure ISQ values and showed that effect magnitude and statistical significance varied across assumptions. Thus, the findings are not robust to all failure-related assumptions and should be interpreted as associations with ISQ trajectories while implants remained in situ, rather than evidence that preoperative aMMP-8 predicts implant failure.

## 5. Conclusions

In this prospective cohort study, higher preoperative salivary aMMP-8 was associated with a less favorable early implant-stability trajectory, with differences emerging at 8 weeks and persisting at 12 weeks, despite little evidence of an association with implant stability at placement. These findings suggest that preoperative salivary aMMP-8 may capture host-response variation associated with early implant healing but do not establish its prognostic utility or a causal role in osseointegration. The clinical significance of the observed ISQ differences remains uncertain. The associations were sensitive to certain assumptions regarding implant failure, and exploratory analyses suggested potential nonlinearity. Furthermore, these associations do not establish clinically actionable aMMP-8 thresholds or justify modifications to conventional implant-loading protocols. External prospective validation and dedicated prediction studies are warranted to determine whether aMMP-8 provides incremental prognostic information beyond established clinical and mechanical factors before its use in clinical risk stratification can be considered.

## Figures and Tables

**Table 1:** Baseline participant, periodontal, salivary and implant characteristics (*N* = 125).

Characteristic	Value
**Participant characteristics**	
Age, years	56.4 ± 10.0
Sex	
Female	67 (53.6)
Male	58 (46.4)
Smoking status	
Never	69 (55.2)
Former	37 (29.6)
Current	19 (15.2)
Smoking pack-years	0.0 [0.0–12.9]
Diabetes	12 (9.6)
HbA1c, %	5.3 [5.1–5.5]
**Periodontal and salivary characteristics**	
Periodontal status	
Periodontal health	43 (34.4)
Gingivitis	33 (26.4)
Treated/stable periodontitis	49 (39.2)
Bleeding on probing, % of sites	8.6 ± 6.1
Plaque, % of sites	21.7 ± 9.5
Salivary flow rate, mL/min	0.39 [0.31–0.49]
Preoperative aMMP-8, ng/mL	142.3 [95.9–223.8]
ln(aMMP-8)	5.01 ± 0.64
**Implant and surgical characteristics**	
Jaw	
Maxilla	65 (52.0)
Mandible	60 (48.0)
Implant location	
Anterior	19 (15.2)
Posterior	106 (84.8)
Bone quality (Lekholm–Zarb)	
D1	6 (4.8)
D2	45 (36.0)
D3	55 (44.0)
D4	19 (15.2)
Bone quality, collapsed	
D1 + D2	51 (40.8)
D3 + D4	74 (59.2)
Implant length, mm	
8.5	18 (14.4)
10.0	47 (37.6)
11.5	36 (28.8)
13.0	24 (19.2)
Implant diameter, mm	
3.5	6 (4.8)
4.0	26 (20.8)
4.5	68 (54.4)
5.0	25 (20.0)
Insertion torque, N cm	36.3 ± 9.3
ISQ at implant placement (T0)	75.9 ± 8.3

Values are mean ± SD, median [IQR] or *n* (%). aMMP-8, active matrix metalloproteinase-8; ISQ, implant stability quotient; IQR, interquartile range. Bone quality was collapsed to D1 + D2 versus D3 + D4 for analysis because of sparse D1 observations. Anterior sites included incisors and canines; posterior sites included premolars and molars, classified using FDI tooth-position codes.

**Table 2:** Observed ISQ and measurement availability at each visit (*N* = 125 participants; 500 scheduled measurements).

	T0	Week 4	Week 8	Week 12
Observed, *n* (%)	125 (100)	120 (96.0)	113 (90.4)	113 (90.4)
Ordinarily missing, *n*	0	3	7	6
Structurally unavailable after failure, *n*	0	2	5	6
Cumulative implant failures, *n*	0	2	5	6
ISQ, mean ± SD	75.9 ± 8.3	69.8 ± 7.5	71.4 ± 6.7	73.8 ± 7.6
ISQ, median [IQR]	76.5 [71.0–81.5]	70.5 [65.0–75.5]	71.5 [67.0–76.0]	74.0 [69.5–79.5]
ISQ, range	50.5–92.5	52.5–86.5	57.0–87.5	52.5–89.5
Change from T0, mean ± SD (*n*)	—	−6.05 ± 5.82 (120)	−4.42 ± 7.09 (113)	−2.21 ± 7.98 (113)

Observed (unadjusted) values. Change from T0 is calculated among participants observed at both T0 and the given visit. Structurally unavailable measurements are scheduled visits occurring after implant failure and are not treated as missing data. Cumulative failures are those occurring on or before the scheduled visit.

**Table 3:** Primary MMRM estimates for the association between preoperative salivary aMMP-8 and ISQ trajectory (125 participants, 471 observations).

Effect	Estimate	SE	95% CI	*p*	Holm *p*	KR df
**Global test (primary hypothesis)**						
aMMP-8 × time (H_0_: γ_4_ = γ_8_ = γ_12_ = 0)	*F*(3, 112.1) = 8.81			<0.001	—	3, 112.1
**Week-specific contrasts (secondary; gated)**						
Week 4 vs. T0 (γ_4_)	−0.81	0.53	−1.85 to 0.23	0.126	0.126	118.9
Week 8 vs. T0 (γ_8_)	−2.55	0.60	−3.75 to −1.35	<0.001	<0.001	113.9
Week 12 vs. T0 (γ_12_)	−3.09	0.66	−4.39 to −1.79	<0.001	<0.001	111.4
**Cross-sectional association at T0 (for context)**						
ln(aMMP-8) main effect	0.30	0.69	−1.07 to 1.66	0.669	—	129.3

Estimates are adjusted differences in mean ISQ change from T0 per 1-SD higher preoperative ln(aMMP-8) (≈1.89-fold higher aMMP-8 concentration). Unstructured covariance, REML, Kenward–Roger degrees of freedom (week 4, 118.9; week 8, 113.9; week 12, 111.4). Adjusted for age, sex, smoking status, diabetes status, periodontal status, salivary flow, jaw, implant length, implant diameter, insertion torque and bone quality (D1 + D2 vs. D3 + D4), with insertion torque × time and bone quality × time interactions. Exact Holm-adjusted *p* values: week 4, 0.126; week 8, 1.02 × 10^−4^; week 12, 2.16 × 10^−5^. Exact global *p* = 2.71 × 10^−5^.

**Table 4:** Preoperative salivary active matrix metalloproteinase-8 concentrations by periodontal status and correlations with periodontal inflammatory parameters.

	Periodontal Health (*n* = 43)	Gingivitis (*n* = 33)	Treated/Stable Periodontitis (*n* = 49)	*p*
aMMP-8, ng/mL, median [IQR]	106.2 [79.6–140.4]	184.2 [132.5–360.3]	158.8 [116.6–222.9]	<0.001 ^a^
aMMP-8, ng/mL, range	31.1–377.6	65.8–771.4	35.3–527.0	
aMMP-8, geometric mean, ng/mL	109.5	214.1	156.8	
Geometric mean ratio vs. health (95% CI)	1 (reference)	1.96 (1.50 to 2.56)	1.43 (1.12 to 1.82)	
Dunn pairwise vs. health, Holm *p*	—	<0.001	0.004	
BOP, % of sites, median [IQR]	2.6 [0.5–8.1]	13.5 [11.4–18.2]	8.0 [4.1–11.1]	
Plaque, % of sites, median [IQR]	15.8 [10.7–21.2]	28.3 [23.6–35.1]	22.3 [18.0–26.2]	
Spearman ρ, aMMP-8 vs. BOP% (95% CI)	0.02 (−0.28 to 0.32)	0.33 (−0.02 to 0.61)	0.54 (0.28 to 0.72)	
Spearman ρ, aMMP-8 vs. plaque% (95% CI)	0.08 (−0.23 to 0.37)	−0.00 (−0.35 to 0.34)	0.26 (−0.02 to 0.51)	
**All participants (*N* = 125)**				
Spearman ρ, aMMP-8 vs. BOP% (95% CI)	0.47 (0.32 to 0.60)			<0.001
Spearman ρ, aMMP-8 vs. plaque% (95% CI)	0.36 (0.20 to 0.51)			<0.001

^a^ Kruskal–Wallis χ^2^(2) = 19.98; *p* < 0.001; Dunn post hoc test for gingivitis vs. treated/stable periodontitis, Holm-adjusted *p* = 0.116. Geometric mean ratios from a linear model of ln(aMMP-8) (*F*(2, 122) = 12.50; *p* < 0.001). Correlation CIs by the Bonett–Wright method. Partial Spearman correlation of aMMP-8 with BOP% adjusted for plaque%: ρ = 0.34 (*p* < 0.001); with plaque% adjusted for BOP%: ρ = 0.10 (*p* = 0.271). BOP, bleeding on probing.

## Data Availability

The datasets generated and analyzed during the current study are available from the corresponding author upon reasonable request.

## Supplementary Materials

The following supporting information can be downloaded at https://www.mdpi.com/article/10.3390/jcm15218650/s1. Table S1. Primary MMRM: fixed effects, covariance, collinearity and functional-form diagnostics (125 participants, 471 observations). Table S2. Specification and results of the primary and sensitivity analyses. Table S3. Periodontal inflammatory burden: additional correlation analyses, periodontally healthy subgroup and effect modification. Table S4. Characteristics of failed (*n* = 6) and surviving (*n* = 119) implants and individual information on the failures. Table S5. Failure-inclusive sensitivity analyses treating implant failure as an unfavorable outcome. Figure S1. Restricted cubic spline analysis (S3b). Difference in mean ISQ change from T0 relative to the median aMMP-8 (142.3 ng/mL) across the 2.5th–97.5th percentile range of aMMP-8 (log scale), from the primary model with a 3-knot restricted cubic spline of ln(aMMP-8) (knots 72.0, 142.3, 363.9 ng/mL) interacting with time; solid line and band, estimate and 95% CI; dashed line, log-linear primary model; rug, observed values. Joint spline × time test *F*(6, 145.97) = 6.28; *p* < 0.001; test of departure from log-linearity (nonlinear spline × time terms) *F*(3, 110.8) = 3.15; *p* = 0.028. Figure S2. Failure-inclusive δ-adjusted tipping-point analysis. Post-failure ISQ values were multiply imputed under missing at random (δ = 0, analysis S11) and then shifted downward by δ ISQ units; points and band show the week-specific differences in ISQ change from T0 per 1-SD higher ln(aMMP-8) with 95% CIs, and the dotted navy line the primary estimate. The global aMMP-8 × time test remained significant through δ = −20 (*F*(3, 116.4) = 2.96; *p* = 0.035) and not at δ = −25 (*F*(3, 116.5) = 2.06; *p* = 0.109). Composite and worst-rank results are given in Table S5.
