# Supplementary material for: Preoperative Salivary Active MMP-8 and Early Dental Implant Stability Trajectories: A Prospective Cohort Study
Source: J Clin Med. 2026 Nov 6;15(21):8650. doi: 10.3390/jcm15218650 (PMC13764060; doi:10.3390/jcm15218650)
Supplement: Supplementary file 1 [file jcm-15-08650-s001.zip › jcm-4624711-supplementary.pdf]

Supplementary Materials

Table S1. Primary MMRM: fixed effects, covariance, collinearity and functional-form diagnostics (125 participants, 471 observations).

A. Fixed-effect estimates

Term	Estimate	SE	KR df	95% CI	*P*
Intercept	60.390	8.210	125.7	44.142 to 76.638	< 0.001
ln(aMMP-8), per 1 SD (effect at T0)	0.296	0.691	129.3	−1.071 to 1.664	0.669
Week 4 (vs T0)	−1.594	2.694	119.9	−6.929 to 3.740	0.555
Week 8 (vs T0)	0.463	3.086	115.4	−5.650 to 6.575	0.881
Week 12 (vs T0)	7.405	3.405	115.1	0.660 to 14.150	0.032
Age, per year	0.005	0.050	108.4	−0.094 to 0.103	0.928
Male sex (vs female)	0.840	0.989	105.6	−1.121 to 2.800	0.398
Former smoker (vs never)	1.100	1.167	104.7	−1.215 to 3.415	0.348
Current smoker (vs never)	−0.801	1.427	105.3	−3.631 to 2.029	0.576
Diabetes (vs no)	−0.808	1.703	103.6	−4.187 to 2.570	0.636
Gingivitis (vs periodontal health)	0.111	1.411	107.3	−2.686 to 2.908	0.937
Treated/stable periodontitis (vs health)	−1.507	1.183	106.2	−3.853 to 0.839	0.206
Salivary flow, per 1 mL/min	4.457	3.272	106.7	−2.030 to 10.944	0.176
Mandible (vs maxilla)	1.483	1.081	105.5	−0.661 to 3.627	0.173
Implant length, per 1 mm	−0.064	0.345	106.5	−0.749 to 0.621	0.854
Implant diameter, per 1 mm	−0.497	1.384	108.6	−3.241 to 2.246	0.720
Insertion torque, per 1 Ncm (effect at T0)	0.444	0.082	130.0	0.283 to 0.606	< 0.001
Bone quality D3+D4 vs D1+D2 (effect at T0)	−0.878	1.518	127.9	−3.880 to 2.125	0.564
ln(aMMP-8) × week 4 (γ4)	−0.811	0.526	118.9	−1.852 to 0.231	0.126
ln(aMMP-8) × week 8 (γ8)	−2.547	0.605	113.9	−3.746 to −1.349	< 0.001
ln(aMMP-8) × week 12 (γ12)	−3.088	0.656	111.4	−4.387 to −1.789	< 0.001
Insertion torque × week 4	−0.111	0.062	119.0	−0.235 to 0.012	0.076
Insertion torque × week 8	−0.138	0.072	115.1	−0.280 to 0.004	0.057
Insertion torque × week 12	−0.242	0.078	113.5	−0.398 to −0.087	0.002
Bone quality D3+D4 × week 4	−0.763	1.169	118.9	−3.077 to 1.552	0.515
Bone quality D3+D4 × week 8	0.077	1.346	114.4	−2.591 to 2.744	0.955
Bone quality D3+D4 × week 12	−1.037	1.488	114.5	−3.985 to 1.911	0.487

Reference levels: T0, female, never smoker, no diabetes, periodontal health, maxilla, bone quality D1+D2. Because of the time interactions, the main effects of ln(aMMP-8), insertion torque and bone quality are their associations with ISQ at T0, and the × week terms are differences in change from T0. The intercept corresponds to all continuous covariates at zero.

B. Estimated unstructured covariance matrix

	T0	Week 4	Week 8	Week 12
T0	51.28	32.70	19.82	21.34
Week 4	*0.664*	47.31	21.01	19.66
Week 8	*0.487*	*0.537*	32.32	17.59
Week 12	*0.450*	*0.432*	*0.467*	43.85
SD	7.16	6.88	5.69	6.62

Variances and covariances on and above the diagonal; correlations below the diagonal (italic). REML log-likelihood −1459.73; 27 fixed-effect and 10 covariance parameters; converged. REML AIC 2939.5 (unstructured) vs 2939.5 (heterogeneous Toeplitz), 2941.9 (heterogeneous compound symmetry) and 2949.2 (heterogeneous AR(1)); see S9a–c in Table S2.

C. Collinearity

Covariate	df	GVIF	$GVIF^{1/(2df)}$	$GVIF^{1/(2df)}$ with BOP% and plaque%
ln(aMMP-8) (z)	1	1.30	1.14	1.25
Age	1	1.10	1.05	1.05
Sex	1	1.10	1.05	1.06
Smoking status	2	1.34	1.08	1.11
Diabetes	1	1.15	1.07	1.10
Periodontal status	2	1.52	1.11	1.27
Salivary flow	1	1.17	1.08	1.09
Jaw	1	1.33	1.15	1.18
Implant length	1	1.31	1.14	1.14
Implant diameter	1	1.25	1.12	1.12
Insertion torque	1	1.58	1.26	1.29
Bone quality (collapsed)	1	1.51	1.23	1.23
BOP%	1	—	—	1.80
Plaque%	1	—	—	1.42

Generalized variance inflation factors for the participant-level covariate design. Condition index of the predictor correlation matrix 2.6. Variance of the exposure contrasts in the adjusted vs unadjusted MMRM: ratio 0.94–0.99. Spearman correlations of ln(aMMP-8) with continuous covariates other than BOP% (0.47) and plaque% (0.36) were between −0.10 and 0.13.

Table S1 (continued).

D. Functional form

Term	Alternative specification	Test vs linear	*P*	ΔAIC	γ_4 / γ_8 / γ_{12}
Age	restricted cubic spline, 3 knots	$\chi^2(1) = 0.20$	.659	1.8	−0.81 / −2.55 / −3.09
Salivary flow	restricted cubic spline, 3 knots	$\chi^2(1) = 1.39$	.238	0.6	−0.81 / −2.55 / −3.09
Insertion torque	restricted cubic spline, 3 knots, × time	$\chi^2(4) = 3.65$	.455	4.3	−0.95 / −2.68 / −3.16
Implant length	categorical (indicator) coding	$\chi^2(2) = 0.75$	.688	3.3	−0.81 / −2.55 / −3.09
Implant diameter	categorical (indicator) coding	$\chi^2(2) = 0.94$	.625	3.1	−0.81 / −2.55 / −3.09
ln(aMMP-8) (exposure)	Restricted cubic spline, 3 knots, × time (S3b)	$F(3, 110.8) = 3.15$	.028	−1.4	Table S2
ln(aMMP-8) (exposure)	Untransformed concentration (S3a; non-nested)	—	—	−5.1	Table S2

Each continuous covariate was respecified one at a time; likelihood-ratio tests from maximum-likelihood fits, γ from the corresponding REML refit (primary: −0.81 / −2.55 / −3.09). For the exposure, the Kenward–Roger test of the nonlinear spline × time terms is shown. ΔAIC < 0 favors the alternative.

Table S2. Specification and results of the primary and sensitivity analyses.

ID	Analysis	Model	Estimand	Exposure	Adjustment	*n* / obs.	Missing data	Global test	Week 4	Week 8	Week 12
**Primary analysis**											
—	Primary analysis	MMRM-UN	Primary[a]	1 SD ln	Full[d]	125 / 471	DL (MAR)	$F(3, 112.1) = 8.81$; $P < 0.001$	−0.81 (−1.85 to 0.23)	−2.55 (−3.75 to −1.35)	−3.09 (−4.39 to −1.79)
**A. Model and exposure specification**											
S1	Constrained baseline	MMRM-UN, T0 effect fixed at 0	Primary[a]	1 SD ln	Full[d]	125 / 471	DL (MAR)	$F(3, 114.3) = 12.63$; $P < 0.001$	−0.72 (−1.68 to 0.24)	−2.39 (−3.36 to −1.43)	−2.94 (−4.05 to −1.83)
S2	Postbaseline ANCOVA *	MMRM-UN, weeks 4–12	Conditional[b]	1 SD ln	Full + T0 ISQ × visit	123 / 346	DL (MAR)	$F(3, 111.2) = 11.95$; $P < 0.001$	−0.78 (−1.79 to 0.24)	−2.46 (−3.48 to −1.44)	−3.01 (−4.15 to −1.86)
S3a	Untransformed aMMP-8 *	MMRM-UN	Primary[a]	1 SD raw (132.7 ng/mL)	Full[d]	125 / 471	DL (MAR)	$F(3, 111.6) = 12.40$; $P < 0.001$	−0.89 (−1.94 to 0.17)	−2.89 (−4.06 to −1.72)	−3.51 (−4.76 to −2.26)
S3b	Restricted cubic spline *	MMRM-UN + RCS	Primary[a]	P75 vs P25[c]	Full[d]	125 / 471	DL (MAR)	$F(6, 146.0) = 6.28$; $P < 0.001$	−0.85 (−2.26 to 0.55)	−2.93 (−4.54 to −1.32)	−3.45 (−5.19 to −1.72)
**B. Covariate adjustment**											
S4a	Covariate set	MMRM-UN	Primary[a]	1 SD ln	Full + BOP%	125 / 471	DL (MAR)	$F(3, 112.1) = 8.81$; $P < 0.001$	−0.81 (−1.85 to 0.23)	−2.55 (−3.75 to −1.35)	−3.09 (−4.39 to −1.79)
S4b1	Covariate set	MMRM-UN	Primary[a]	1 SD ln	Full; BOP% replaces periodontal status	125 / 471	DL (MAR)	$F(3, 112.1) = 8.72$; $P < 0.001$	−0.81 (−1.85 to 0.23)	−2.54 (−3.74 to −1.34)	−3.07 (−4.37 to −1.77)
S4b2	Covariate set	MMRM-UN	Primary[a]	1 SD ln	Full; BOP% + plaque% replace periodontal status	125 / 471	DL (MAR)	$F(3, 112.3) = 8.73$; $P < 0.001$	−0.82 (−1.86 to 0.23)	−2.54 (−3.74 to −1.34)	−3.08 (−4.38 to −1.78)
S4c	Covariate set	MMRM-UN	Primary[a]	1 SD ln	Full; pack-years replace smoking status	125 / 471	DL (MAR)	$F(3, 111.9) = 8.76$; $P < 0.001$	−0.81 (−1.85 to 0.23)	−2.54 (−3.74 to −1.34)	−3.09 (−4.39 to −1.79)
S4d	Minimally adjusted	MMRM-UN	Primary[a]	1 SD ln	Age, sex, torque × time, bone quality × time[e]	125 / 471	DL (MAR)	$F(3, 111.8) = 8.70$; $P < 0.001$	−0.80 (−1.85 to 0.24)	−2.54 (−3.75 to −1.33)	−3.08 (−4.39 to −1.78)
S4e	Covariate set	MMRM-UN	Primary[a]	1 SD ln	Full; torque and bone quality without × time	125 / 471	DL (MAR)	$F(3, 114.4) = 8.06$; $P < 0.001$	−0.79 (−1.84 to 0.25)	−2.54 (−3.75 to −1.33)	−3.00 (−4.34 to −1.66)
S4f	Covariate set	MMRM-UN	Primary[a]	1 SD ln	Full; HbA1c replaces diabetes	125 / 471	DL (MAR)	$F(3, 112.2) = 8.82$; $P < 0.001$	−0.81 (−1.85 to 0.23)	−2.55 (−3.74 to −1.35)	−3.09 (−4.39 to −1.79)
S4g	Covariate set †	MMRM-UN	Primary[a]	1 SD ln	Full + BOP% + plaque%	125 / 471	DL (MAR)	$F(3, 112.2) = 8.78$; $P < 0.001$	−0.82 (−1.86 to 0.22)	−2.54 (−3.74 to −1.35)	−3.08 (−4.38 to −1.78)
**C. Sample, missing data and influence**											
S5	Survivors through week 12	MMRM-UN	Primary[a], survivors	1 SD ln	Full[d]	119 / 460	DL; 6 failed implants excluded	$F(3, 109.9) = 8.51$; $P < 0.001$	−1.10 (−2.15 to −0.04)	−2.61 (−3.82 to −1.39)	−3.10 (−4.40 to −1.79)
S6	Complete cases †	MMRM-UN	Primary[a], complete cases	1 SD ln	Full[d]	106 / 424	Complete case (19 excluded)	$F(3, 100.0) = 6.46$; $P < 0.001$	−0.87 (−1.97 to 0.22)	−2.55 (−3.84 to −1.27)	−2.73 (−4.14 to −1.32)
S7	Multiple imputation	MMRM-UN, pooled	Primary[a]	1 SD ln	Full[d]	125 / 487	MI, PMM, m = 50[f]	$F(3, 112.9) = 8.28$; $P < 0.001$	−0.91 (−1.96 to 0.14)	−2.44 (−3.65 to −1.22)	−3.09 (−4.39 to −1.79)
S8	Influential participants	MMRM-UN	Primary[a]	1 SD ln	Full[d]	121 / 455	DL; 4 influential excluded[g]	$F(3, 109.1) = 11.17$; $P < 0.001$	−0.87 (−2.02 to 0.28)	−2.71 (−4.02 to −1.41)	−3.92 (−5.31 to −2.53)
S8b	Influential participants (4/n rule) †	MMRM-UN	Primary[a]	1 SD ln	Full[d]	117 / 441	DL; 8 influential excluded[g]	$F(3, 106.1) = 8.39$; $P < 0.001$	−1.64 (−2.88 to −0.39)	−2.76 (−4.20 to −1.32)	−3.73 (−5.24 to −2.21)
**D. Covariance structure**											
S9a	Heterogeneous Toeplitz †	MMRM-TOEPH	Primary[a]	1 SD ln	Full[d]	125 / 471	DL (MAR)	$F(3, 153.2) = 8.80$; $P < 0.001$	−0.82 (−1.97 to 0.33)	−2.56 (−3.77 to −1.35)	−3.05 (−4.36 to −1.74)
S9b	Heterogeneous compound symmetry †	MMRM-CSH	Primary[a]	1 SD ln	Full[d]	125 / 471	DL (MAR)	$F(3, 230.3) = 10.54$; $P < 0.001$	−0.85 (−2.08 to 0.38)	−2.54 (−3.71 to −1.37)	−3.03 (−4.27 to −1.79)
S9c	Heterogeneous AR(1) †	MMRM-AR(1)H	Primary[a]	1 SD ln	Full[d]	125 / 471	DL (MAR)	$F(3, 219.5) = 6.14$; $P < 0.001$	−0.79 (−1.93 to 0.35)	−2.58 (−3.92 to −1.23)	−3.08 (−4.66 to −1.50)

a Primary estimand: difference in mean ISQ change from T0 per exposure unit while the implant remained in situ; post-failure measurements were structurally unavailable. S5 and S6 estimated the same contrast in restricted populations.

b S2: difference in postbaseline ISQ per 1 SD ln(aMMP-8), conditional on T0 ISQ; global test assesses the exposure effect across follow-up visits (3 df).

c S3b: 75th-versus-25th percentile contrast (223.8 vs 95.9 ng/mL; ≈1.33 SD ln[aMMP-8]); spline knots at 72.0, 142.3, and 363.9 ng/mL. Global spline × time test: 6 df; departure from log-linearity: $F(3, 110.8) = 3.15$, $P = 0.028$. Corresponding primary-model contrasts at weeks 4, 8, and 12: −1.08 (−2.47 to 0.31), −3.39 (−4.99 to −1.80), and −4.11 (−5.85 to −2.38), respectively (Figure S1).

d Full adjustment: age, sex, smoking, diabetes, periodontal status, salivary flow, jaw, implant length and diameter, insertion torque × time, and bone quality × time.

e S4d adjustment: age, sex, insertion torque × time, and bone quality × time.

f S7: multiple imputation by chained equations using predictive mean matching (5 donors; 50 imputations; 20 iterations; seed 2024). Only 16 intermittently missing ISQ values were imputed; post-failure observations were excluded. Predictors included exposure, primary covariates, and ISQ at other visits. Estimates were pooled using Rubin's rules with Barnard–Rubin degrees of freedom; global testing used D1 with Reiter degrees of freedom. Fractions of missing information were 0.050, 0.019, and 0.002 at weeks 4, 8, and 12. Estimates were stable across five seeds and an alternative Bayesian regression imputation model.
g S8: leave-one-participant-out generalized Cook's distance identified influential observations. S8 excluded the four largest (P007, P118, P036, P065); S8b excluded eight participants exceeding 4/n = 0.032 (additionally P076, P037, P004, P104).
Abbreviations: DL, direct likelihood under missing at random; MI, multiple imputation; ISQ, implant stability quotient. Week-specific sensitivity estimates are unadjusted for multiplicity; Holm adjustment applies to the primary analysis (Table 3).
***** S2 estimates a conditional postbaseline association, whereas S3a uses standardized untransformed aMMP-8 (1 SD = 132.7 ng/mL). S3b uses a percentile contrast. These estimates are not directly comparable in magnitude with the primary estimate.
Failure-inclusive sensitivity analyses (S10–S12) are presented in Table S5.

Table S3. Periodontal inflammatory burden: additional correlation analyses, periodontally healthy subgroup and effect modification.

A. Correlation analyses not shown in Table 4

**Analysis**	***n***	**ρ (95% CI)**	***P***
BOP% vs plaque%	125	0.62 (0.49 to 0.73)	< 0.001
Partial: aMMP-8 vs BOP%, adjusted for plaque%	125	0.34 (0.17 to 0.49)	< 0.001
Partial: aMMP-8 vs plaque%, adjusted for BOP%	125	0.10 (−0.08 to 0.27)	0.271

Spearman and partial rank correlations (partial: Pearson correlation of rank residuals; Fisher-z CI). Overall and within-category correlations of aMMP-8 with BOP% and plaque% are reported in Table 4. Proportion of variance in ln(aMMP-8) explained: periodontal status 17.0%; BOP% alone 28.6%; periodontal status, BOP% and plaque% together 30.1%.

B. Periodontally healthy subgroup and effect modification by periodontal status

**Analysis**	**Participants / obs.**	**Global aMMP-8 × time**	**Week 4**	**Week 8**	**Week 12**
Full cohort, primary model (reference)	125 / 471	$F(3, 112.1) = 8.81$; $P < 0.001$	−0.81 (−1.85 to 0.23)	−2.55 (−3.75 to −1.35)	−3.09 (−4.39 to −1.79)
Health, fully adjusted[a]	43 / 153	$F(3, 32.8) = 1.04$; $P = 0.389$	0.05 (−2.30 to 2.41)	−1.43 (−3.67 to 0.80)	−2.12 (−5.00 to 0.76)
Health, parsimonious[b]	43 / 153	$F(3, 31.9) = 1.18$; $P = 0.334$	0.04 (−2.34 to 2.43)	−1.45 (−3.75 to 0.85)	−2.42 (−5.35 to 0.52)
Health, survivors, fully adjusted[a]	37 / 142	$F(3, 30.7) = 0.97$; $P = 0.421$	−0.84 (−3.32 to 1.63)	−1.57 (−3.89 to 0.76)	−2.43 (−5.34 to 0.49)
**Group-specific estimates, aMMP-8 × time × periodontal status model (full cohort)**					
Periodontal health	125 / 471	—	−0.17 (−2.29 to 1.96)	−1.44 (−4.02 to 1.14)	−2.55 (−5.30 to 0.20)
Gingivitis	125 / 471	—	−2.23 (−4.13 to −0.33)	−4.46 (−6.61 to −2.32)	−5.30 (−7.61 to −3.00)
Treated/stable periodontitis	125 / 471	—	−0.49 (−2.44 to 1.47)	−1.01 (−3.15 to 1.14)	−1.44 (−3.77 to 0.88)
Test of effect modification (6 df)	125 / 471	$F(6, 141.1) = 1.28$; $P = 0.269$	—	—	—

[a] Primary covariates without periodontal status (22 fixed effects for 43 participants). [b] Insertion torque × time and bone quality × time only. The healthy subgroup contains all six failed implants. SD of ln(aMMP-8) was 0.52 in the healthy subgroup vs 0.64 overall; estimates remain per 1 cohort SD.

Table S4. Characteristics of failed (n = 6) and surviving (n = 119) implants and individual information on the failures.

A. Failed versus surviving implants

Characteristic	Failed (n = 6)	Surviving (n = 119)	SMD	*P*
Age, years	59.5 [50.5–61.8]	56.0 [50.0–64.0]	−0.21	0.768
Female sex	3/6 (50)	64/119 (53.8)	−0.08	1.000
Current smoker	1/6 (17)	18/119 (15.1)	0.04	1.000
Diabetes	1/6 (17)	11/119 (9.2)	0.22	0.461
Periodontal health	6/6 (100)	37/119 (31.1)	2.11	0.001
BOP, %	3.1 [0.5–8.7]	8.8 [3.2–12.3]	−0.72	0.070
Plaque, %	16.6 [11.1–24.1]	21.9 [16.2–27.2]	−0.57	0.265
aMMP-8, ng/mL	105.3 [70.7–109.2]	151.5 [97.8–229.3]	−1.04	0.015
Salivary flow, mL/min	0.38 [0.33–0.43]	0.39 [0.30–0.49]	0.08	0.782
Maxilla	3/6 (50)	62/119 (52.1)	−0.04	1.000
Bone quality D3+D4	3/6 (50)	71/119 (59.7)	−0.20	0.687
Implant length, mm	10.0 [10.0–11.1]	10.0 [10.0–11.5]	−0.40	0.365
Implant diameter, mm	4.0 [4.0–4.4]	4.5 [4.5–4.5]	−0.52	0.126
Insertion torque, Ncm	27.9 [27.0–31.4]	36.6 [29.6–44.0]	−1.08	0.030
ISQ at T0	70.0 [63.9–73.9]	77.5 [71.2–81.8]	−0.50	0.154

Median [IQR] with Wilcoxon rank-sum *P*, or *n* (%) with Fisher exact *P*. SMD, standardized mean difference (failed − surviving). No failures occurred among participants with gingivitis (0/33) or treated/stable periodontitis (0/49). Descriptive comparisons; *P* values are unadjusted and not intended for inference given six events.

B. Individual failed implants (ordered by time to failure)

ID	Age/sex	aMMP-8, ng/mL (cohort percentile)	Jaw/site (FDI), bone	Torque, Ncm	ISQ T0 / W4 / W8 / W12	Failure, week	Reason
P052	65/F	104.5 (30)	Mandible/46, D2	27.1	91.0 / — / — / —	2.8	Early mobility/loss of stability
P096	61/M	111.8 (33)	Maxilla/17, D3	21.2	59.5 / — / — / —	3.7	Failure of osseointegration
P010	58/F	110.2 (32)	Maxilla/15, D3	27.0	73.5 / 65.5 / — / —	4.3	Failure of osseointegration
P115	31/M	106.2 (31)	Mandible/35, D2	35.6	66.5 / 68.5 / — / —	6.2	Early mobility/loss of stability
P076	48/F	43.3 (3)	Mandible/46, D2	32.3	74.0 / 59.5 / — / —	6.9	Failure of osseointegration
P058	62/M	59.4 (5)	Maxilla/24, D4	28.6	63.0 / 52.5 / 62.5 / —	11.5	Failure of osseointegration

All six participants were periodontally healthy; one was a current smoker and one had diabetes. — not measured (structurally unavailable after failure). Cohort median aMMP-8, 142.3 ng/mL. Failure proportion 4.8% (exact 95% CI 1.8%–10.2%).

Table S5. Failure-inclusive sensitivity analyses treating implant failure as an unfavorable outcome.

A. ISQ trajectory: composite and hypothetical strategies

Strategy	Participants / obs.	Global aMMP-8 × time	Week 4	Week 8	Week 12
Primary (implant in situ; post-failure visits not used)	125 / 471	$F(3, 112.1) = 8.81$; $P < 0.001$	−0.81 (−1.85 to 0.23)	−2.55 (−3.75 to −1.35)	−3.09 (−4.39 to −1.79)
S10a Composite: post-failure ISQ = lowest observed ISQ at that visit	125 / 484	$F(3, 116.8) = 3.85$; $P = 0.011$	−0.67 (−1.83 to 0.50)	−2.09 (−3.40 to −0.78)	−2.10 (−3.63 to −0.58)
S10b Composite: lowest observed ISQ − 10	125 / 484	$F(3, 117.2) = 2.04$; $P = 0.112$	−0.58 (−1.85 to 0.68)	−1.78 (−3.26 to −0.31)	−1.66 (−3.41 to 0.10)
S11 Hypothetical: post-failure ISQ imputed, δ = 0	125 / 500	$F(3, 116.3) = 8.05$; $P < 0.001$	−0.95 (−2.01 to 0.11)	−2.41 (−3.63 to −1.19)	−3.09 (−4.41 to −1.77)
Tipping point, δ = −5	125 / 500	$F(3, 116.3) = 6.79$; $P < 0.001$	−0.91 (−1.98 to 0.16)	−2.24 (−3.48 to −1.01)	−2.86 (−4.19 to −1.54)
Tipping point, δ = −10	125 / 500	$F(3, 116.3) = 5.41$; $P = 0.002$	−0.86 (−1.95 to 0.23)	−2.08 (−3.35 to −0.80)	−2.64 (−4.00 to −1.27)
Tipping point, δ = −15	125 / 500	$F(3, 116.3) = 4.09$; $P = 0.008$	−0.82 (−1.94 to 0.30)	−1.91 (−3.24 to −0.58)	−2.41 (−3.84 to −0.99)
Tipping point, δ = −20 (S11b)	125 / 500	$F(3, 116.4) = 2.96$; $P = 0.035$	−0.78 (−1.94 to 0.39)	−1.74 (−3.15 to −0.34)	−2.19 (−3.69 to −0.69)
Tipping point, δ = −25	125 / 500	$F(3, 116.5) = 2.06$; $P = 0.109$	−0.73 (−1.94 to 0.48)	−1.58 (−3.08 to −0.08)	−1.96 (−3.56 to −0.36)
Tipping point, δ = −30	125 / 500	$F(3, 116.5) = 1.39$; $P = 0.249$	−0.69 (−1.95 to 0.58)	−1.41 (−3.01 to 0.19)	−1.74 (−3.44 to −0.03)
Tipping point, δ = −40	125 / 500	$F(3, 116.6) = 0.57$; $P = 0.633$	−0.60 (−2.00 to 0.80)	−1.08 (−2.92 to 0.76)	−1.29 (−3.25 to 0.67)

Composite: post-failure visits assigned the lowest ISQ observed in the cohort at that visit (week 4, 52.5; week 8, 57.0; week 12, 52.5) or 10 units lower; ordinarily missing values by direct likelihood. Hypothetical/tipping point: all 29 missing ISQ values multiply imputed under MAR (MICE-PMM, m = 50, seed 2024), imputed post-failure values shifted by δ, primary MMRM fitted and pooled by Rubin's rules (D1 global test). See Figure S2.

B. Worst-rank composite (S12)

Week	Analysis	*n*	OR per 1-SD ln(aMMP-8) (95% CI)	*P*
Week 8	Worst-rank composite	125	0.66 (0.46 to 0.94)	0.023
Week 8	Survivors only, failures excluded	120	0.58 (0.40 to 0.85)	0.005
Week 12	Worst-rank composite	125	0.51 (0.35 to 0.74)	< 0.001
Week 12	Survivors only, failures excluded	119	0.40 (0.26 to 0.59)	< 0.001

ISQ change from T0 ranked, with failed implants ranked below all survivors (earlier failure worse); proportional-odds regression adjusted for the primary covariates, pooled over the 50 imputations of S7. OR < 1 indicates a less favorable rank with higher aMMP-8. Exploratory event model (6 events; not adequately powered or adjusted): Firth logistic regression, OR for failure per 1-SD ln(aMMP-8) 0.35 (0.13 to 0.86) ($P = 0.020$); with insertion torque added, 0.35 (0.12 to 0.86).

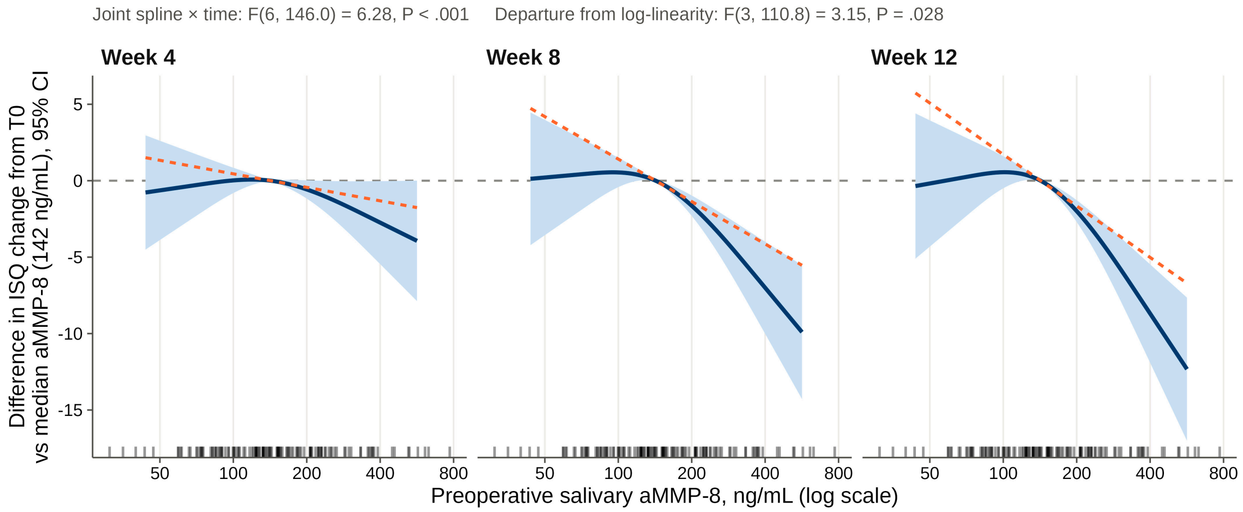

Figure S1. Restricted cubic spline analysis (S3b). Difference in mean ISQ change from T0 relative to the median aMMP-8 (142.3 ng/mL) across the 2.5th–97.5th percentile range of aMMP-8 (log scale), from the primary model with a 3-knot restricted cubic spline of ln(aMMP-8) (knots 72.0, 142.3, 363.9 ng/mL) interacting with time; solid line and band, estimate and 95% CI; dashed line, log-linear primary model; rug, observed values. Joint spline × time test $F(6, 145.97) = 6.28$; $P < 0.001$; test of departure from log-linearity (nonlinear spline × time terms) $F(3, 110.8) = 3.15$; $P = 0.028$.

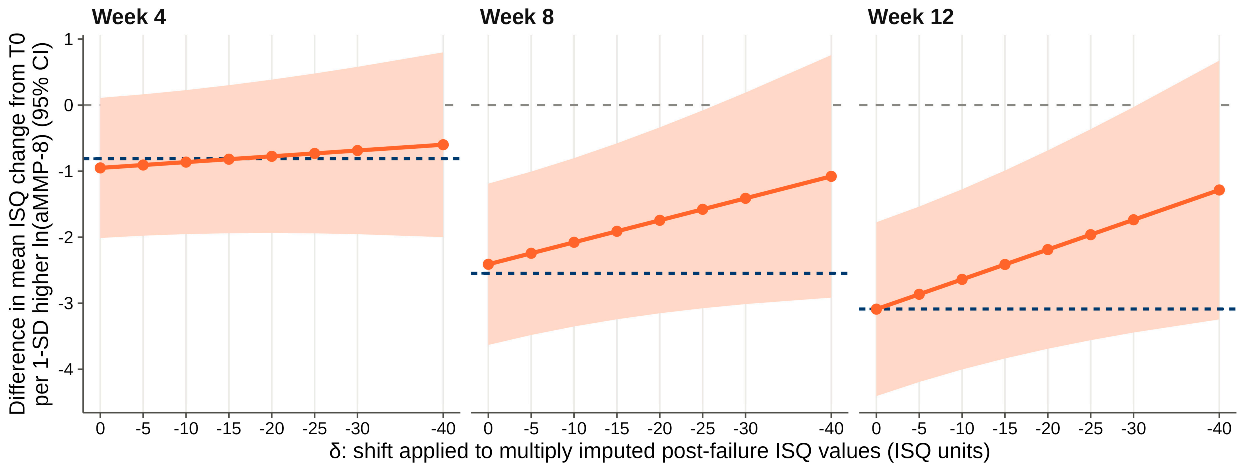

Figure S2. Failure-inclusive δ-adjusted tipping-point analysis. Post-failure ISQ values were multiply imputed under missing at random (δ = 0, analysis S11) and then shifted downward by δ ISQ units; points and band show the week-specific differences in ISQ change from T0 per 1-SD higher ln(aMMP-8) with 95% CIs, and the dotted navy line the primary estimate. The global aMMP-8 × time test remained significant through δ = −20 ($F(3, 116.4) = 2.96$; $P = 0.035$) and not at δ = −25 ($F(3, 116.5) = 2.06$; $P = 0.109$). Composite and worst-rank results are given in Table S5.
